# Presence and Causes of Sterilization Equipment Failures with Biological Indicators in Dental Offices in Mexico: A Longitudinal Cohort

**DOI:** 10.3390/medicina60091525

**Published:** 2024-09-19

**Authors:** Nuria Patiño-Marín, Lorena Dafnee Villa-García, Yolanda Terán-Figueroa, Carlo Eduardo Medina-Solis, Yesica Yolanda Rangel-Flores, Marco Felipe Salas-Orozco, Fidel Martínez-Gutiérrez, Eva Concepción Aguirre-López

**Affiliations:** 1 Clinical Research Laboratory, Faculty of Stomatology, Autonomous University of San Luis Potosí, San Luis Potosi 78290, Mexico; lorena.vill@uaslp.mx (L.D.V.-G.); marco-sala@hotmail.com (M.F.S.-O.); evguirrelopez@hotmail.com (E.C.A.-L.); 2 Faculty of Nursing and Nutrition, Autonomous University of San Luis Potosí, San Luis Potosi 78290, Mexico; yolanda@uaslp.mx (Y.T.-F.); yesica.range@uaslp.mx (Y.Y.R.-F.); 3 Academic Area of Dentistry of Health Sciences Institute, Autonomous University of Hidalgo State, Pachuca 42160, Mexico; cemedinas@yahoo.com; 4 Faculty of Chemical Sciences, Autonomous University of San Luis Potosí, San Luis Potosi 78290, Mexico; fidl@uaslp.mx

**Keywords:** sterilization, dental practices, biological indicators

## Abstract

*Background/Objectives*: Sterilization in dental practice is crucial for infection prevention. The aim of this study was to identify the presence and causes of bacterial growth using biological indicators in dental sterilization equipment in San Luis Potosí, S.L.P., Mexico, with different consecutive measurements over a year. *Methods*: This longitudinal cohort, conducted from January 2022 to January 2024 in San Luis Potosí, Mexico, aimed to identify the presence and causes of bacterial growth in dental sterilization equipment using biological indicators. A total of 207 dental offices were approached, and 175 participated, providing data through questionnaires and monitoring sterilization cycles with BIs. The checks were bimonthly for one year, with a total of six checks. *Results*: (a) An 11% (*n* = 1188) incidence of bacterial growth was observed, with a higher percentage in dry heat equipment (13%). (b) Upon analyzing the six consecutive verifications over a year, no statistically significant differences were observed in the failures of the sterilization cycles when comparing the two pieces of equipment. (c) Error in temperature and time of the equipment (OR = 4.0, 95% CI = 1.6–3.9, *p* = 0.0001) was significantly associated with the presence of bacterial growth during the one-year period. *Conclusions*: Monitoring sterilization cycles and identifying the causes of bacterial growth with different consecutive verifications decreased the presence of bacterial growth (failures) during the one-year period.

## 1. Introduction

Sterilization is crucial in dental practices for infection prevention and the safety of patients and staff. It involves steam autoclaves and dry heat sterilizers to remove pathogens from instruments. Effectiveness is monitored through the use of external and internal chemical indicators and biological indicators (BIs), with BIs being the gold standard as they can be used to test for the elimination of resistant bacterial spores. Despite their importance, routine BI monitoring in Mexico is not widespread, though it is recommended by health authorities [1,2]. Most studies published on sterilization cycle failures have been cross-sectional studies; however, these have certain limitations, such as the inability to establish causal relationships, often incurring prevalence bias, and not capturing the temporal variability of exposures and outcomes [2,3,4,5].

Longitudinal studies are noted for their ability to track variables and outcomes over time, providing valuable insight into continuous processes like sterilization in dental practice. This methodology is essential for understanding trends and patterns that could influence sterilization efficacy, allowing for the identification of recurrent issues and failure factors in steam autoclaves and dry heat sterilizers [6].

The limited use of longitudinal studies in dental sterilization research has left a gap in understanding the factors influencing sterilization efficacy over time. Therefore, this study aimed to identify the presence and causes of bacterial growth using biological indicators in dental sterilization equipment in San Luis Potosí, S.L.P., Mexico, with different consecutive measurements over a year [5,7].

## 2. Materials and Methods

A longitudinal cohort with consecutive measurements was conducted from January 2022 to January 2024 at the Autonomous University of San Luis Potosí. This study adhered to ethical standards and ensured informed consent under the Declaration of Helsinki. It received approval from the Research Ethics Committee of the Faculty of Stomatology (CEIFE-407. Approval Date: January 2022).

### 2.1. Study Population

**Figure 1:**
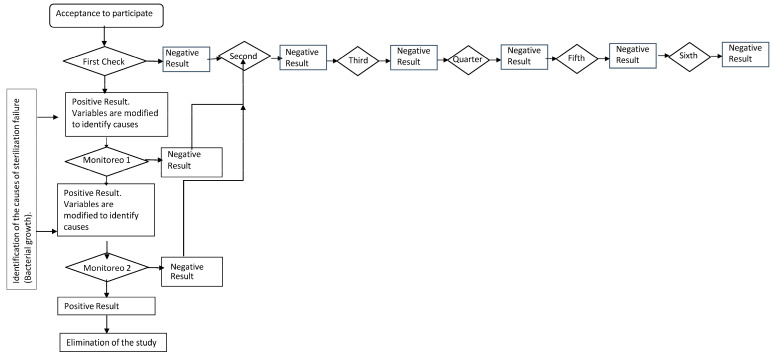
Procedure and number of checks of the sterilization equipment with biological indicators from the longitudinal study.

The selection of the dental offices was carried out through non-probability consecutive sampling. From 207 dental offices across five zones in San Luis Potosí, Mexico, 175 subjects owning a dental office met the following selection criteria: (a) for inclusion, (1) both genders, (2) ages 24–66, and (3) operators possessing sterilization equipment, either dry heat or autoclave. The exclusion criteria included the following: (a) individuals who did not provide written consent to participate; (b) sterilizers not conducting the sterilization cycle; and (c) dental offices not located in the central, northern, eastern, western, and southern zones of the City of San Luis Potosí (State of San Luis Potosí, Mexico). The elimination criteria were as follows: (a) undelivered or incomplete questionnaires, (b) contaminated samples, and (c) three consecutive positive results from the same sterilization equipment during checks (Figure 1).

Participants followed a protocol that included the following:

1. *Questionnaire*: Information was collected about the type of sterilizer and management, adherence to the manufacturer’s guidelines, daily sterilization cycles, operator responsibility, use of biological indicators, and maintenance.
2. *Monitoring of Sterilization Cycles*:
  (a) *Biological Indicators*: This study utilized *Bacillus atrophaeus* spore strips (Sporigam Biological Indicator, Gamma Biolabs, CDMX, Mexico City, Mexico) to monitor dry heat equipment and *Geobacillus stearothermophilus* spores (Sporigam Biological Indicator, Gamma Biolabs, CDMX, Mexico) were used for autoclaves were used, which were placed in the sterilization equipment for a standard cycle.
  (b) *Sample Processing*: The culture medium employed to detect the presence or absence of bacterial growth was Soy Trypticase (BD Bioxon, Becton Dickinson de Mexico, Estado de Mexico, Mexico City, Mexico) with 0.25% anhydrous dextrose (Hycel de México, S.A de C.V., México D.F., Roma Norte, Mexico). Three milliliters of this culture medium was added to each tube (sample), and the tubes were then incubated at 37 °C for 7 days to assess dry heat sterilization cycles, and at 57 °C for 7 days to evaluate autoclaves. A positive control (indicating bacterial growth), a negative control (indicating the absence of bacterial growth), and a culture medium control were utilized for each sample. To confirm the results of the cultures, each of the samples, including the controls, was seeded onto Soybean–Casein Digest Agar (BD Bioxon, Becton Dickinson de Mexico, Estado de Mexico, Mexico) after 48 h and re-incubated for an additional 48 h at 37 °C for dry heat sterilization cycles and at 57 °C for autoclaves [5,8,9,10].
  (c) *Results Interpretation*: Tests determined the presence (equipment failures) or absence of bacterial growth, indicating the performance of the sterilizer.

*Procedure and Frequency of Checks*. The checks were bimonthly and followed the NOM 013 standard for one year, with a total of 6 checks. Negative results allowed participants to continue in the study, while positive results prompted an analysis of the causes of such outcomes (Figure 1).

For the identification of causes in this study, each detection of bacterial growth involved two monitoring phases to identify, record, and correct equipment malfunctions. Adjustments were made to the operation of the sterilizer after the first positive detection. If the first monitoring showed ongoing issues (positive result), additional adjustments were made, and a second monitoring was conducted. A second positive result led to the removal of the equipment from the study, while a negative result indicated a successful correction, allowing the equipment to continue in the study (Figure 1).

### 2.2. Statistical Analysis

The kappa simple test was performed to standardize the investigators in the variable of the presence or absence of bacterial growth. In the univariate analysis, categorical variables were reported with frequencies and percentages; the continuous variables were expressed with means and standard deviations. The distribution of the data (continuous variables) was tested by means of Shapiro–Wilk and Brown–Forsythe tests. We used the Chi-square test to establish the differences between the presence or absence of bacterial growth and the questionnaire at the time of each verification. A binary logistic regression multivariate analysis was performed to estimate associations. In the analysis, the presence of bacterial growth (presence of growth in some verification of the six measurements) or absence of growth was established as a dependent dichotomous variable. The independent variables were a correct or incorrect procedure, the use of biological indicators, and the maintenance of the equipment. (a) A variance inflation factor (VIF) analysis test was conducted to detect and mitigate multicollinearity among the independent variables; (b) the specification error test verified the assumption that the response variable’s logit is a linear combination of the independent variables; (c) interactions were tested and none were significant; and (d) the model’s overall fit was assessed using the goodness of fit test. The association between dependent and independent variables is presented as odds ratios (ORs) with 95% confidence intervals (CIs) [11]. *p* values < 0.05 were deemed to be statistically significant. The analysis was performed using JMP version 10.0 (SAS Institute, Cary, NC, USA) and Stata version 11.0 (Stata Corp LP, College Station, TX, USA).

## 3. Results

A total of 207 dentists with practices established in the City of San Luis Potosí were invited to participate. In total, 175 (85%) dentists (female = 105 (60%), male = 70 (40%)) ranging in age from 24 to 66 years old (mean = 38 ± 10), agreed to participate in the study. Only 32 (15%) dental surgeons declined for the following reasons: (a) they were not interested (*n* = 25), (b) their equipment was not functioning (*n* = 5), and (c) such verifications were conducted elsewhere (*n* = 2). There were 210 sterilization units involved in the study; 132 (63%) were autoclaves (operating time in minutes: 33 ± 17; temperature: 130 °C ± 19; and pressure: 25 psi ± 12) and 78 (37%) were dry heat (operating time in minutes: 60 ± 23; temperature: 180 °C ± 35), distributed across five zones of the City of San Luis Potosí (*n* = 28 units from the Centro zone, *n* = 55 from the Norte, *n* = 36 from the Oriente, *n* = 32 from the Poniente, and *n* = 59 from the Sur). Throughout the study, 18 units were removed due to showing three consecutive results with bacterial growth during verification (Figure 1).

In Table 1, the presence and absence of bacterial growth in the autoclave and dry heat equipment across six verifications are presented. An 11% (*n* = 128) incidence of bacterial growth was identified out of all evaluations (*n* = 1188), with a higher percentage found in the dry heat equipment (13%, *n* = 56 units). When comparing both types of equipment between all the verifications, statistically significant differences were only observed during the first verification (*p* < 0.0010), where a 30% incidence of bacterial growth was noted in dry heat units. Analyzing the six consecutive verifications (bimonthly) of each unit over a year, 106 (55%) units exhibited no growth, whereas 86 (45%) showed at least one positive result during all evaluations.

The association between questionnaire responses and the presence or absence of bacterial growth across six verifications is detailed in Table 2. Factors such as (a) training of personnel using the equipment, (b) equipment maintenance, (c) correct equipment procedure, and (d) receipt of information from biological indicators were associated with the absence of bacterial growth (*p* < 0.005) in the various verifications across all sterilization equipment (*n* = 1188). Regarding the use of biological indicators, only 10% (*n* = 19) of participants reported using them monthly or bimonthly.

Table 3 and Table 4 identify the results of bacterial growth across six verifications and two incidences of monitoring for each verification in both autoclave and dry heat equipment. Concerning the autoclave equipment (Table 3), considering the six verifications and two incidences of monitoring, the most frequent cause of bacterial growth were errors in temperature and the time of the first verification (*n* = 8, 53%). After conducting Monitoring 1 and 2 to reduce bacterial growth, it was observed that the frequency of growth in the equipment decreased across all verifications, with technical failure becoming the most common cause in the last incidence of monitoring (Monitoring 2: *n* = 5, 83%) during the first verification. Regarding dry heat equipment (Table 4), the most frequent cause of bacterial growth was temperature and/or time errors during the first verification (*n* = 11, 48%). After conducting various monitoring sessions, the frequency of bacterial growth decreased across all verifications, and like autoclave sterilizers, the most frequent cause in Monitoring 2 was technical failure (*n* = 3, 60%) during the first verification. 

In the multivariate analysis using binary logistic regression, the independent variable of an incorrect procedure (OR = 4.0, 95% CI = 1.6–3.9, *p* = 0.0001) was significantly associated with the presence of bacterial growth during the six verifications.

## 4. Discussion

In our study, 175 of 207 dentists participated, achieving an 85% participation rate, which is significant for a longitudinal cohort given the usual challenges, such as attrition and the high costs associated with maintaining large sample sizes over time. Longitudinal studies offer advantages like tracking long-term changes and identifying causal relationships in sterilization cycle efficacy within dental clinics, thus enhancing the quality of data despite potential difficulties in participant engagement and representativeness [6,12,13].

Furthermore, the participation rate in our cohort (85%) exceeds that typically reported in previous studies on the same topic, ranging from approximately 47% to 60% [4,9]. For example, a study conducted in Iran reported the participation of 84 dental clinics but did not report the total population sampled [14]. A study conducted in the United Kingdom focused on assessing knowledge about autoclave use and sterilization routines among dentists, reporting a participation rate of 47.7% [4]. Another study conducted in Norway, which evaluated failures in sterilization cycles in 1985 and 1991, reported a participation rate of 60%. It is important to note that, unlike previous studies, ours is a longitudinal cohort, and to our knowledge, previous studies on the topic have been cross-sectional in design. The higher participation rate in our study compared to similar studies from other countries can be attributed to several factors, including a strong researcher–participant relationship built through clear communication and trust, as well as local cultural and institutional support that encouraged engagement. This study’s direct relevance to participants’ daily practices, particularly regarding sterilization and patient safety, likely motivated their involvement. The high participation rate had several valuable implications for the outcomes of this study. First, the high participation rate reduced the risk of selection bias, making the sample more representative of the broader population of dental offices. This enhanced the generalizability of the findings across various settings, including differences in practice size, location, and equipment types. Second, with a larger and more engaged sample, the statistical power of this study was increased, allowing for more precise estimates and robust conclusions. The ability to detect significant associations and trends was enhanced, providing stronger evidence for identifying factors that contributed to sterilization failures and the effectiveness of different sterilization methods [15]. 

In this study, by comparing only the results from the first verification, we found statistically significant differences (*p* = 0.0010) between wet and dry heat sterilizers, identifying higher bacterial growth in dry heat sterilizers (30%, *n* = 23). This result aligns with many previous cross-sectional studies [12]. However, upon analyzing the six consecutive verifications performed on both wet and dry heat sterilizers over a year, a slightly higher percentage of sterilization cycle failures was observed in dry heat sterilizers (13%) compared to wet heat sterilizers (10%); however, this difference was not significant. This appears to contradict the outcomes of previous cross-sectional studies, which suggest that dry heat leads to a significantly higher presence of failures. This was likely due to the more stringent requirements for achieving effective sterilization with dry heat, which involves higher temperatures and longer exposure times. These requirements make dry heat sterilization more prone to human error, such as incorrect temperature settings, improper loading, or uneven heat distribution, especially when operators are not adequately trained or the equipment is not properly maintained. Consequently, the initial discrepancies in bacterial growth between the methods could be attributed to these challenges. However, over time, our study found no significant differences in bacterial growth between dry and wet heat sterilization methods, suggesting that with consistent use, regular equipment maintenance, and comprehensive training, the performance of dry heat sterilizers can be comparable to that of wet heat sterilizers. This convergence in efficacy over time implies that the initial higher bacterial growth rates with dry heat sterilizers were likely due to a learning curve or initial lapses in maintenance and adherence to protocols. Our study adds new insights by demonstrating that the effectiveness of dry heat sterilization can improve significantly when operators become more familiar with the equipment and ensure that it is regularly calibrated and maintained [16]. Therefore, the choice between them should not be based on the efficiency reported in previous cross-sectional articles but on other factors, such as dry heat being ideal for metal instruments that could be damaged by moisture, but not suitable for those with plastic or rubber components, as they may deform [17]. In terms of efficiency and time, dry heat tends to be slower and less efficient in heat transfer, while autoclaves are faster due to the penetration of steam under pressure. From a cost and maintenance perspective, dry heat can be more economical initially and, in its upkeep, but less energy-efficient in the long run, while autoclaves, though more expensive, justify their expenditure through efficiency in high-demand settings. Regarding space and convenience, dry heat sterilizers are generally smaller and more convenient for clinics with limited space, while larger autoclaves are essential in practices with a high volume of instruments. Wet heat sterilization, such as autoclaving, though initially more expensive due to high equipment and maintenance costs, proves more cost-effective in high-volume settings, like hospitals and busy dental clinics, where quick turnaround times are crucial. The faster cycles of autoclaves, their versatility in terms of different materials, and built-in validation features make them a reliable choice that minimizes the cost of potential failures. In contrast, dry heat sterilization is more economical for settings with fewer sterilization needs and materials that are heat-resistant and moisture-sensitive. It has lower upfront and maintenance costs and is suitable for a broader range of non-aqueous materials. However, its longer cycle times and potential for higher energy consumption can be drawbacks. In resource-limited settings, where financial and infrastructure constraints exist, dry heat sterilization may be a more feasible option despite these limitations [18]. Local regulations on infection control also play a role in the choice of sterilization method. Moreover, safety is an important factor; autoclaves pose risks when operating with high-pressure steam, and dry heat sterilizers require caution to avoid burns [19].

According to the results of this study, the total percentage of failures in sterilization devices (both wet and dry heat) was 11%. This outcome is akin to previous studies, such as the one conducted by Gio et al., which reported a failure rate of 7.4% [7]. In a study conducted by Dagher et al., a general failure rate of 10.7% was reported [20]. Research conducted by Vier-Pelisser et al. in Brazil documented a failure rate of 35% in sterilizers [21]. Lastly, a study conducted by Patiño- Marìn et al. indicated a failure rate of 17% [9]. The differences in the failure rates reported in this study compared to those from previous research might be attributable to various study designs (cross-sectional or longitudinal), sample sizes, and methodologies employed.

Upon analyzing the six consecutive verifications, considering both dry heat and wet heat sterilizers, we found that 103 (49%) units showed at least one positive result during the six measurements. There are no previous longitudinal articles reporting a similar percentage, except the study conducted by Acosta et al. in 2002, which reported a total of 242 failures out of 3277 verifications over six years, representing a 7.4% failure rate in sterilization cycles. The differences between our results and those of Acosta et al. may be due to the methodology used. The authors did not specify the sampling time, and some samples took up to 10 days to process as they were sent by mail [7]. 

Sterilization verifications occur bimonthly in Mexico, adhering to national standards, while the U.S. recommends weekly verifications, reflecting different regulatory and resource landscapes. The discrepancy arises from diverse health priorities, resource limitations, and practice environments. Despite these differences, both approaches aim at ensuring patient safety and controlling infections, tailored to each country’s specific capabilities and health governance frameworks [22,23,24].

In the analysis of the causes of sterilization cycle failures, the factors associated with the absence of bacterial growth during verification were (a) the training of personnel in the use of the equipment, (b) equipment maintenance, (c) execution of the correct equipment procedure, and (d) receipt of information from biological indicators. The results of this study highlight that sterilization failures often stem from inadequate staff training [5] with other factors like the incorrect equipment use and insufficient maintenance, underlining the critical need for comprehensive training and proper sterilization practices to ensure effective infection control [4,15,25,26].

This study found that temperature and timing errors were the main causes of bacterial growth in both autoclave and dry heat sterilizers across six verifications and two monitoring procedures. Efforts to reduce failures led to a decrease in bacterial growth frequency, with technical failures becoming the predominant issue. The persistence of certain bacteria and viruses at temperatures up to 60 °C highlights the importance of maintaining adequate temperature and duration in sterilization processes to ensure microbial deactivation [27,28,29]. Some studies also indicate that the wrapping material for instruments can impact the temperature achieved during sterilization, linking to technical failures and bacterial growth observed in this study’s verifications [30].

Temperature and timing errors, which were the primary causes of sterilization failures, often arise from several underlying issues related to operators, equipment, maintenance, and environmental conditions. Operator error is a significant factor, frequently resulting from inadequate training or failure to adhere to sterilization protocols. Mistakes such as incorrect settings for temperature and time, improper loading techniques, or prematurely interrupted cycles can compromise sterilization effectiveness. Addressing these issues requires comprehensive and ongoing training programs that focus on proper equipment use, understanding critical sterilization parameters, and regular competency assessments to reinforce correct practices. Equipment malfunctions also contribute to sterilization failures, often due to wear and tear, improper maintenance, or calibration issues. Problems such as faulty heating elements, damaged seals, or inaccurate sensors can prevent sterilizers from reaching or maintaining necessary temperatures. Regular maintenance and calibration schedules, with checks of critical components and adjustments by qualified technicians, are essential to prevent such malfunctions. Inadequate maintenance itself is another cause; without regular cleaning, filter changes, and component inspections, debris, dust, or mineral deposits can accumulate, impairing sterilizer performance. Establishing preventive maintenance protocols, maintaining detailed logs, and training staff to recognize early signs of malfunction can help mitigate these risks. Additionally, environmental factors, such as room temperature, humidity, and air circulation, can affect sterilization cycles and the drying phase, potentially leading to incomplete sterilization or conditions that promote microbial growth. Controlling environmental conditions by using climate control systems, monitoring with thermometers and hygrometers, and ensuring that sterilization occurs in suitable settings can help maintain the efficacy of sterilization processes. Altogether, reducing temperature and timing errors requires a multifaceted approach that includes operator training, routine equipment maintenance, and environmental controls to ensure reliable and effective sterilization, thereby improving infection control and patient safety in healthcare settings [31].

The longitudinal design allowed us to track changes in sterilization practices, equipment performance, and the occurrence of sterilization failures over an extended period. This approach enabled us to identify patterns and trends that are not discernible in a cross-sectional study, which provides only a snapshot at a single point in time. By following the same dental offices over time, we were able to observe the cumulative effects of interventions, equipment maintenance, and changes in operator behavior on sterilization outcomes.

Furthermore, the longitudinal nature of this study provided insights into the temporal relationship between the implementation of training programs, equipment maintenance schedules, and improvements or failures in sterilization practices. This time-dependent analysis was crucial for understanding the effectiveness and sustainability of strategies aimed at reducing sterilization failures, which cannot be adequately captured in cross-sectional research [32].

The findings of our study emphasize the critical need for regular sterilization cycle checks in dental practices to ensure effective infection control by identifying and addressing any errors or equipment malfunctions that could compromise instrument sterility. This is especially important in resource-limited settings, where challenges such as outdated equipment, insufficient training, and limited maintenance capabilities heighten the risk of sterilization failures. Therefore, it is essential for policymakers to implement guidelines that mandate regular sterilization checks and periodic audits while also providing accessible training and maintenance resources. Such policies should promote the development and distribution of low-cost, easy-to-maintain sterilization equipment and establish regional centers or mobile units for equipment servicing and operator training. By adopting these measures, this manuscript highlights a comprehensive approach to improving sterilization practices and patient safety, particularly in under-resourced environments [33].

## 5. Conclusions

(a) An 11% (*n* = 1188) incidence of bacterial growth was observed, with a higher percentage in dry heat equipment (13%). (b) Upon analyzing the six consecutive verifications over a year, no statistically significant differences were observed in the failures of the sterilization cycles when comparing the two pieces of equipment. (c) Error in temperature and time of the equipment (OR = 4.0, 95% CI = 1.6–3.9, *p* = 0.0001) was significantly associated with the presence of bacterial growth over a one-year period.

## Figures and Tables

**Table 1:** Presence or absence of bacterial growth in sterilization equipment: autoclave and dry heat over 6 checks.

Equipment	Autoclave						Dry Heat
	Frequency (%)						
Checks	Presence	Absence	Total	Presence	Absence	Total	*p*
First (*n* = 210)	15 (11)	117 (89)	132	23 (30)	55 (70)	78	0.0010 *
Second (*n* = 200)	9 (7)	118 (93)	127	4 (5)	69 (95)	73	0.6533
Third (*n* = 198)	18 (14)	107 (86)	125	8 (10)	65 (90)	73	0.4843
Fourth (*n* = 195)	15 (12)	109 (88)	124	4 (5)	67 (95)	71	0.1431
Fifth (*n* = 193)	11 (9)	112 (91)	123	12 (17)	58 (83)	70	0.0970
Sixth (*n* = 192)	4 (3)	118 (97)	122	5 (7)	65 (93)	70	0.2330
Total (*n* = 1188)	72 (10)	681 (90)	753	56 (13)	379 (87)	435	0.0507

Statistical test: chi-square (X^2^). * Statistically significant differences (*p* < 0.005).

**Table 2:** Association between survey responses and the presence or absence of bacterial growth over 6 checks of the study equipment.

Questions (Number of Checks)	Presence	Absence	Total	*p*
1. Was the staff trained regarding the use of the equipment? (First check)				
Yes	22	135	157	
Total	38	172	210	0.0087
2. Does the equipment receive maintenance? (First and sixth check)				
Yes	24	188	212	
Total	47	355	402	0.0001
3. Equipment procedure. (First, second, third, fifth, and sixth check)				
Correct	42	704	746	
Total	109	884	993	0.0001
4. Did you receive information about biological indicators? (Fourth check)				
Yes	18	124	142	
Total	19	176	195	0.0091

Statistical test: chi-square (X^2^).

**Table 3:** Bacterial growth results over 6 checks and two incidences of monitoring for each check of autoclave equipment.

Checks	First	Monitoring 1	Monitoring 2	Second	Monitoring 1	Monitoring 2	Third	Monitoring 1	Monitoring 2
Causes					Frequency (%)				
Error in temperature or time	3 (20)	2 (26)	--	--	--	--	7 (39)	--	--
Error in temperature and time	8 (53)	--	1 (17)	5 (56)	1 (25)	--	7 (39)	1 (50)	--
No maintenance	3 (20)	2 (24)	--	2 (22)	--	--	1 (6)	--	--
Inadequate IB handling	1 (7)	4 (50)	--	1 (11)	--	--	2 (10)	--	--
Technical failure	--	--	5 (83)	1 (11)	3 (75)	1 (100)	1 (6)	1 (50)	1 (100)
Total	15 (100)	8 (100)	6 (100)	9 (100)	4 (100)	1 (100)	18 (100)	2 (100)	1 (100)
	**Fourth**	**Monitoring 1**	**Monitoring 2**	**Fifth**	**Monitoring 1**	**Monitoring 2**	**Sixth**	**Monitoring 1**	**Monitoring 2**
Error in temperature or time	3 (20)	--	--	--	--	--	--	--	--
Error in temperature and time	6 (40)	1 (50)	--	4 (36)	2 (40)	--	--	--	--
No maintenance	2 (13)	--	--	1 (10)	1 (20)	--	--	--	1 (100)
Inadequate IB handling	2 (13)	--	--	4 (36)	1 (20)	1 (100)	--	--	--
Technical failure	2 (13)	1 (50)	1 (100)	2 (18)	1 (100)	--	3 (100)	1 (100)	--
Total	15 (100)	2 (100)	1 (100)	11 (100)	5 (100)	1 (100)	3 (100)	1 (100)	1 (100)

*n* = 100 checks and monitoring procedures.

**Table 4:** Bacterial growth results over 6 checks and two monitoring procedures for each check in dry heat equipment.

Checks	First	Monitoring 1	Monitoring 2	Second	Monitoring 1	Monitoring 2	Third	Monitoring 1	Monitoring 2
Causes					Frequency (%)				
Error in temperature or time	11 (48)	2 (24)	--	1 (25)	0 (50)	--	6 (74)	--	--
Error in temperature and time	11 (48)	2 (24)	1 (20)	3 (75)	0 (50)	--	1 (13)	--	--
No maintenance	1 (4)	1 (13)	1 (20)	--	--	--	1 (13)	--	--
Technical failure	--	3 (39)	3 (60)	--	--	--	--	2 (100)	2 (100)
Total	23 (100)	8 (100)	5 (100)	4 (100)	0 (100)		8 (100)	2 (100)	2 (100)
	**Fourth**	**Monitoring 1**	**Monitoring 2**	**Fifth**	**Monitoring 1**	**Monitoring 2**	**Sixth**	**Monitoring 1**	**Monitoring 2**
Error in temperature or time	1 (25)	--	--	6 (50)	--	--	--	--	--
Error in temperature and time	3 (75)	1 (50)	--	3 (25)	--	--	1 (20)	--	--
Inadequate IB handling	--	--	--	--	--	--	1 (20)	--	--
Technical failure	--	1 (50)	1 (100)	3 (25)	1 (100)	0 (100)	3 (60)	1 (100)	1 (100)
Total	4 (100)	2 (100)	1 (100)	12 (100)	1 (100)	0 (100)	5 (100)	1 (100)	1 (100)

*n* = 79 checks and monitoring procedures.

## Data Availability

The data supporting the reported results can be obtained from the corresponding author.
